# Eosinophilic fasciitis following COVID-19: A case series of 3 patients

**DOI:** 10.1016/j.jdcr.2023.11.019

**Published:** 2023-12-06

**Authors:** Yiwen Li, Ha Eun Kong, Justin Cheeley

**Affiliations:** a Department of Dermatology, Emory University School of Medicine, Atlanta, Georgia; b Department of General Internal Medicine, Emory University School of Medicine, Atlanta, Georgia

**Keywords:** COVID-19, eosinophilic fasciitis, SARS-CoV-2

## Introduction

Eosinophilic fasciitis (EF) is a rare autoimmune connective tissue disorder characterized by inflammation and fibrosis of the fascia.^1^ Sudden or gradual onset of erythematous, tender, symmetric edema followed by plate-like induration in the distal limbs is the typical clinical presentation.^2^ Affected tissues later become bound down and display perifollicular dimpling.

The “groove sign” is a characteristic finding of EF, consisting of a depression along the course of the superficial veins, more marked on elevation of the affected limb.^2^ Peripheral eosinophilia, elevated sedimentation rate, and hypergammaglobulinemia are usually observed, along with elevated serum aldolase and type III procollagen peptide levels reflecting disease activity. Some authors have proposed diagnostic criteria for EF which consists of 1 major and 2 minor criteria. The major criteria include the presence of symmetric plate-like sclerotic lesions on the limbs, the absence of Raynaud phenomenon, and the exclusion of systemic sclerosis. The minor criteria include thickening of the fascia with eosinophilic and monocytic cellular infiltrates, and fascial thickening as evidenced on magnetic resonance imaging.^3^ Severity classification is based on the extent of joint contracture, limited movement, and progression of symptoms.

Although the etiology of EF remains elusive, some potential triggers have been identified, including strenuous exercise,^1^ infectious pathogens such as *Borrelia burgdorferi*,^4^ hematologic disorders, autoimmune diseases, radiotherapy, and exposure to medications such as statins, ramipril, or immune checkpoint inhibitors.^5^ Additionally, there have been several case reports published linking the development of EF to severe acute respiratory syndrome coronavirus 2 (SARS-CoV-2) vaccination.^6^^,^^7^ To the best of our knowledge, there have not been any previous reports of EF associated with SARS-CoV-2 infection in the peer-reviewed literature. Here, we present 3 cases of patients who developed EF after coronavirus disease-2019 (COVID-19).

## Case report

### Patient 1

**Fig 1:**
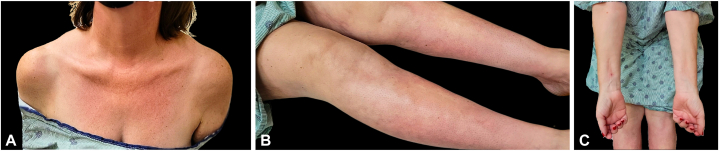
Hyperpigmented plaques on **(A)** superior aspect of the chest, **(B)** poster and lateral lower portion of the legs, **(C)** depression along the course of the superficial veins of right ventral forearms.

A 49-year-old woman with Ehlers-Danlos syndrome first sought medical attention for symptoms of forearm and calf/shin edema, tightness, and tenderness 3 weeks following initial diagnosis of COVID-19. The patient’s condition worsened upon reinfection with SARS-CoV-2 2 months later, and she began to experience exacerbated symptoms such as fatigue, lethargy, skin plaques, tenderness, and diminished range of motion in the wrists and ankles. A positive “groove sign” was identified on the ventral forearms. Furthermore, hyperpigmented bound down plaques were noted on the forearms, thighs, legs, ankles, superior aspect of the chest, left side of the neck, right side of the breast, and upper and lower portion of the abdomen (Fig 1). Notably, there was a decrease in wrist extension upon arm extension and a positive prayer sign, characterized by an inability to fully appose the fingers when the wrists were extended. Following initial mild SARS-CoV-2 infection, laboratory testing revealed an increased peripheral eosinophil count (15%, 1.2 [0.0 – 0.4 × 10^3^/μL]) along with anemia and thrombocytosis. An initial punch biopsy failed to sample the fascia, leading to an inconclusive result. However, magnetic resonance imaging of the tibia/fibula identified marrow and muscular edema along with T2 fascial enhancement. Upon initiating a regimen of prednisone 60 mg per day, the patient experienced a notable alleviation of several symptoms such as fatigue and tightness. However, her condition worsened upon gradual tapering off corticosteroid use, actuating the introduction of methotrexate 10 mg per week. Despite this, the patient experienced progression of bound down, fibrotic plaques, particularly involving the torso and neck.

### Patient 2

**Fig 2:**
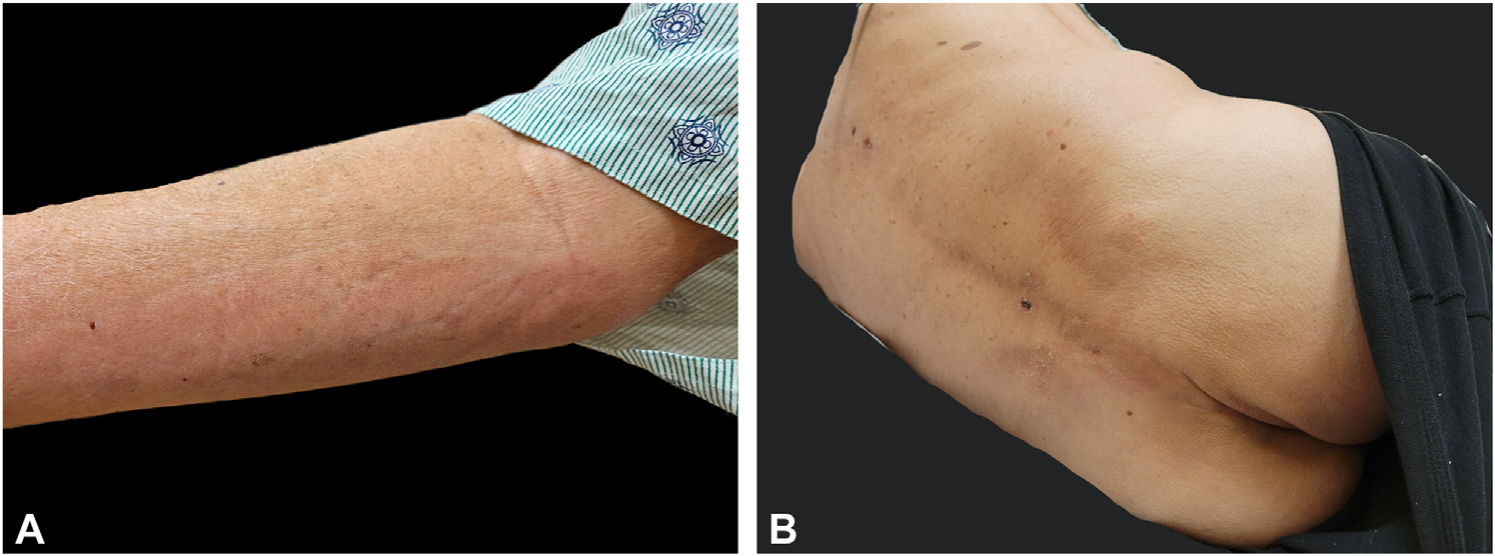
**A,** Depression along the course of the superficial veins of the right ventral forearm; **B,** hyperpigmented plaques on the lower back.

**Fig 3:**
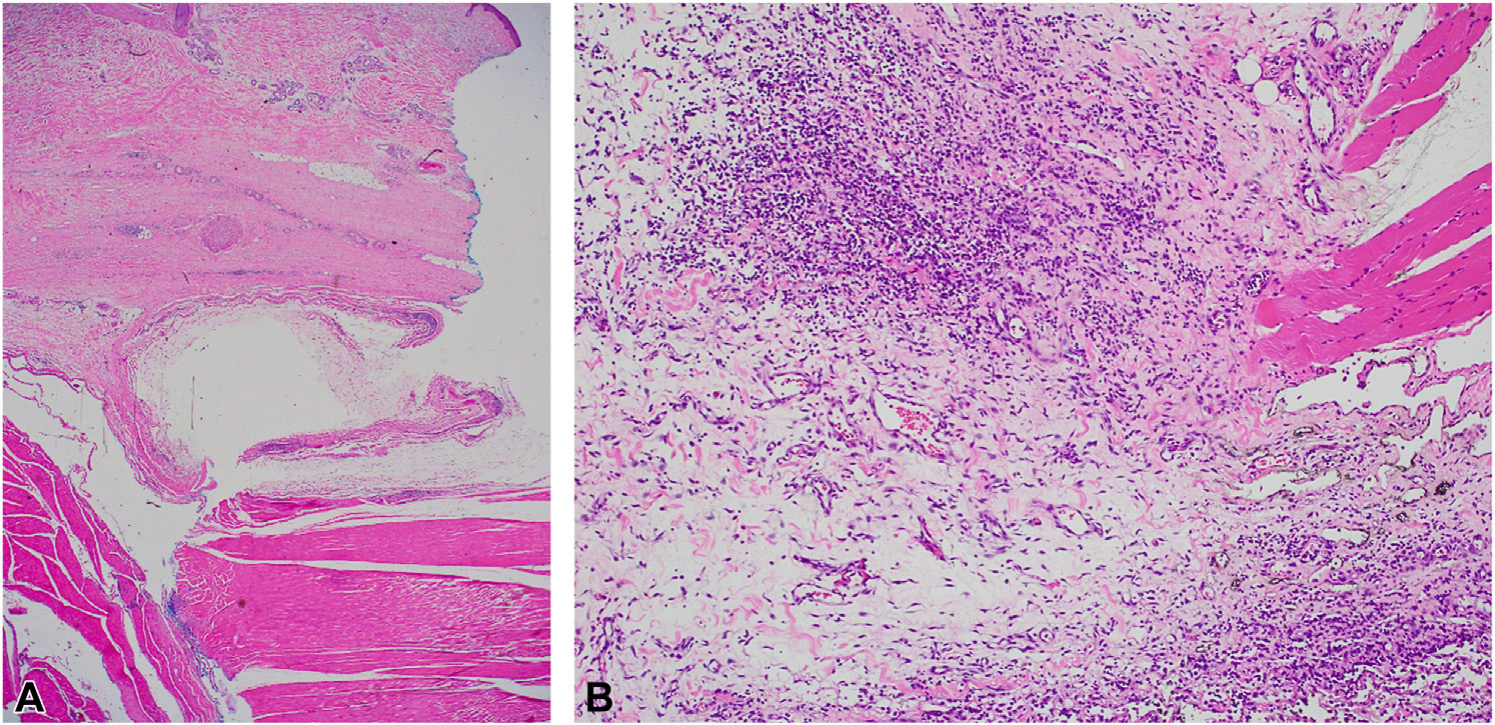
**A,** Diffuse subcutaneous and fascial sclerosis (hematoxylin-eosin stain; original magnification, ×10). **B,** Moderate, mixed inflammatory infiltrate involving the fascia and skeletal muscle with associated edema (hematoxylin-eosin stain; original magnification, ×40).

A 68-year-old man with a medical history of osteoarthritis presented with progressive worsening muscle pain, joint stiffness, fatigue, exertional dyspnea, and swelling in the digits, forearms, ankles, arms, and legs, 7 weeks after SARS-CoV-2 infection. Initially, his symptoms were considered to be manifestations of postacute COVID syndrome, prompting a cardiopulmonary work-up, which returned normal. On physical examination, the patient had difficulty fully extending or flexing his fingers. There was no cuticular dystrophy or nailfold capillary loop dilatation. The patient exhibited taut, bound down, erythematous, shiny plaques affecting the forearms, arms, lower back, buttocks, calves, ankles, and feet. Linear depressions following the course of the veins were evident on the bilateral ventral forearms (Fig 2). On the palmar hands, pink to pale plaques were observed, with accentuation of the eccrine ostia. Laboratory testing revealed an eosinophil count of 7% (0.52 [0.00 – 0.36 × 10^3^/μL]) and a telescoping 10 mm punch biopsy from left forearm revealed diffuse subcutaneous and fascial sclerosis, accompanied by a moderate lymphoplasmacytic inflammatory infiltrate with rare eosinophils (Fig 3). The patient was diagnosed with EF and subsequently managed with prednisone 15 mg per day and methotrexate 20 mg per week concurrently, with great improvement in the range of motion and reduction in edema.

### Patient 3

A 56-year-old woman, with a complex medical history comprising gastroesophageal and intestinal dysmotility necessitating total parenteral nutrition, rectal adenocarcinoma, primary hyperparathyroidism, osteoporosis, and melanoma, developed eosinophilia (10%, 0.71 [0.00 – 0.36 × 10^3^/μL]), paresthesia, edema, progressive induration of the extremities with restricted motion, and low-grade fever with progressive weight loss 2 months after contracting SARS-CoV-2. She reported no recent alterations in her total parenteral nutrition formulations. Physical examination showed plaque formation and stiffness affecting the hips, pelvic girdle, lower thoracic back, bilateral proximal thighs, distal legs, and forearms, with a positive “groove sign.” A skin biopsy revealed a moderate, mixed inflammatory infiltrate affecting the fascia and skeletal muscle, with associated edema. Magnetic resonance imaging findings showed fascial enhancement without involvement of the subcutaneous fat or musculature. The patient was diagnosed with EF and received prednisone 10 mg per day with concurrent methotrexate at 10 mg per week. Six weeks later, dupilumab was introduced at a weekly subcutaneous dose of 300 mg, and prednisone and methotrexate doses were then tapered. The patient reported a significant reduction in edema and substantial improvement in range of motion.

## Discussion

The etiology of EF is complex, with many factors potentially influencing its development. Although some triggers have been identified, the complete pathophysiologic landscape remains elusive. None of the 3 patients described in this case series had a history of preceding exercise, gadolinium exposure within 6 months of EF onset, comorbid Raynaud phenomenon, or symptoms compatible with lichen sclerosis. A noticeable pattern from our case series is the temporal correlation between onset of EF and COVID-19, spanning weeks to several months postinfection. Although this association could be coincidental, the incidence of such a rare disorder post-SARS-CoV-2 infection in 3 separate cases suggests a relationship worthy of further investigation.

Moreover, there have been documented cases of EF emerging after administration of SARS-CoV-2 vaccination. One case report described a 55-year-old patient who developed EF 1 week after receiving the first dose of Vaxzevria SARS-CoV-2 vaccine.^6^ In addition, a 72-year-old woman with a previous history of morphea limited to the abdomen developed EF with skin thickening on the arms and legs 4 weeks after receiving the second dose of Pfizer-BioNTech SARS-CoV-2 vaccine.^7^ Other reported cases have also described patients developing generalized morphea 7 to 20 days after SARS-CoV-2 vaccination^8^ or COVID-19^9^ disease. Although it may be argued that these symptoms following SARS-CoV-2 vaccination or infection could potentially represent an exacerbation of preexisting conditions, there are cases where individuals without prior conditions have also developed EF.^6^ This offers a compelling clue regarding the potential association between EF and COVID-19.

In the 3 cases outlined above, each patient exhibited mild COVID-19 symptoms and did not require hospitalization. Notably, patient 1 and 2 received the last dose of the Moderna vaccine 5 and 11 months, respectively, before contracting COVID-19, followed by the onset of EF. Patient 3 received the Pfizer bivalent booster 3 weeks before COVID-19 and the onset of EF symptoms occurred 2 months after. For patient 1 and 2, given that EF symptoms arose temporally closer to their SARS-CoV-2 infection, it is reasonable to infer that the onset of EF was more likely triggered by COVID-19 than by the vaccination. For patient 3, pinpointing the clear trigger for EF would be complex, given the close interval between her SARS-CoV-2 vaccination and subsequent infection.

The mechanisms by which SARS-CoV-2 might catalyze the onset of EF are multifarious, potentially encompassing direct viral-induced effects, immune dysregulation, and molecular mimicry.^10^ Direct effects of the virus could initiate local tissue damage and inflammation, instigating a cascade of immune responses that eventually manifest as fibrotic alterations, characteristic of EF. Furthermore, the severe systemic inflammatory response, commonly referred to as cytokine storm, that often accompanies COVID-19 could potentially disrupt the finely balanced immune regulation and tolerance mechanisms, leading to an autoimmune reaction. The principle of molecular mimicry posits an additional mechanism through which EF could be triggered.^10^ This principle describes a situation where the immune response, although targeting viral proteins, may inadvertently affect self-antigens in the fascia that share antigenic structures with these proteins, leading to an autoimmune reaction. Viruses, including SARS-CoV-2, are known to induce significant immune dysregulation, resulting in heightened inflammatory responses and imbalances in various immune cell regulations. This disruption could potentially compromise immune tolerance to self-antigens, setting the stage for the emergence of autoimmune conditions such as EF.^10^ Lastly, the concepts of bystander activation and epitope spreading, where a robust immune response to a viral infection inadvertently activates autoreactive immune cells or exposes previously concealed self-antigens, are plausible.^11^ Further research is necessary to solidify these hypotheses and elucidate the precise mechanisms underpinning the association between COVID-19 and EF.

The mechanisms underlying the onset of EF often involve immune dysregulation, marked by eosinophilia. Intriguingly, in hospitalized patients with COVID-19, eosinopenia has emerged as a notable observation. Interpretations of its significance differ among studies, many of which are region-specific. Sun et al found a pronounced decrease in eosinophil counts among patients with severe COVID-19 symptoms, hinting that eosinopenia could be associated with increased disease severity.^12^ The underlying causes of eosinopenia are still debated and could be multifaceted. The possible triggers range from interruptions in the eosinophil life cycle, apoptosis triggered by type 1 interferon during the acute phase of the infection, eosinophil consumption due to their antiviral activities, to reflection of host immune exhaustion.^13^ Notably, the use of corticosteroids, one of the main drugs utilized in the treatment of severe COVID-19, could play a role in the development of eosinopenia.^14^ As patients approached discharge, eosinophil counts generally normalized, indicating that a rebound in these counts might correlate with a patient’s clinical improvement.^15^ This resurgence in eosinophil activity during recovery prompts speculation regarding its potential role in predisposing individuals to conditions such as EF.

Although the link between SARS-CoV-2 vaccination and EF has previously been described, to our knowledge, this case series is the first report of the potential association between COVID-19 and EF. A larger patient cohort and a more exhaustive molecular study are necessary to confirm a temporal relationship between EF and COVID-19.

## Conflicts of interest

None disclosed.
